# mRNA-seq analysis to enhance drought resilience by superabsorbent polymer seed coatings on maize

**DOI:** 10.1186/s13104-025-07621-5

**Published:** 2026-01-27

**Authors:** Akram Abdolmaleki, Hendrik Bertram, Susann Michanski, Armin O. Schmitt, Mehmet Gültas

**Affiliations:** 1 Georg-August University, Margarethe Von Wrangell-Weg 7, 37075 Gottingen, Germany; 2 South Westphalia University of Applied Sciences, Lubecker Ring 2, 59494 Soest, Germany

**Keywords:** SAP, Zea mays L, Germination, Seed coating, Abiotic stress, Drought-regulated genes, Transcriptome, mRNA-seq analysis

## Abstract

**Objectives:**

A persistent and intensifying drought is severely hampering the growth of essential staple crops. While superabsorbent polymers (SAPs) offer a short-term solution to alleviate drought stress, the underlying molecular mechanisms in maize remain largely unexplored. This study addresses this critical research gap by pioneering mRNA sequencing to comprehensively investigate SAPs’ molecular and biological effects in maize, providing the first detailed transcriptomic understanding of their drought-mitigating roles and offering a valuable public dataset for plant breeding.

**Result:**

The current research presents 10 mRNA sequencing samples from maize seedlings across five treatment groups: two fossil-based SAPs (MERCK, SWT), one natural-based SAP (ABG), a drought-stressed control (CS), and a well-watered control (CN). Seedlings were treated for seven days under International Seed Testing Association (ISTA [1]) protocols. For mRNA-seq, indexed cDNA libraries were sequenced on the Illumina NovaSeq X Plus platform. The dataset includes two biological replicates per group, is publicly available in the European Nucleotide Archive (ENA) under accession ERP180397, and represents the first public mRNA-seq dataset to explore SAP-mediated drought resilience in maize.

## Objective

Maize (*Zea mays* L.) is a major global cereal crop, serving as a staple food and a vital source of feed and biofuel for an expanding global population [2–4]. However, maize productivity is severely constrained by both biotic and abiotic stresses [5, 6]. Among these, drought is particularly detrimental, often reducing yields by over 50% due to maize’s high sensitivity to water deficits [4, 5, 7]. The germination and seedling stages represent a critical window of vulnerability for maize, which is more sensitive to drought than most other cereals during this early developmental phase [4]. Successful germination and seedling establishment are crucial for yield potential and are highly dependent on water availability [8, 9]. Fluctuations in seed moisture directly affect the integrity and function of essential macromolecules such as starch and proteins, thereby altering enzymatic processes vital for metabolic activation [9]. The severity of drought stress and the plant’s genetic makeup dictate the extent of detrimental effects [10]. Safeguarding this stage is thus essential for successful crop establishment and productivity. The increasing severity of drought under climate change underscores the urgent need for adaptive management strategies to strengthen crop resilience [11].

SAPs offer a promising solution to mitigate early drought stress [12–16]. These hydrophilic polymers, containing functional groups such as hydroxyl and carbonyl, can absorb and retain water hundreds to thousands of times their own weight, acting as localized reservoirs in the rhizosphere [13–16]. While fossil- and natural-based SAPs differ in properties and efficiency [16], their underlying molecular mechanisms in maize, especially under drought conditions, remain poorly understood. This data note provides the first publicly available mRNA-seq dataset designed to elucidate these mechanisms, offering a unique resource for understanding how different SAP types influence maize’s transcriptomic responses to drought.

## Data description

This data note provides the mRNA-seq dataset obtained from maize seedlings subjected to SAP seed coatings under drought stress. The dataset consists of 10 raw FASTQ files of mRNA reads, representing five distinct treatment groups: MERCK, SWT, ABG, a stressed control (CS), and a well-watered control (CN). All files are publicly available in ENA (http://identifiers.org/ena.embl:ERP180397). The maize cultivar *Zea mays* L. subsp. *indentata (Sturtev.) Zhuk.*, accession number *ZEA 3639*, spring type, was provided by the Leibniz Institute of Plant Genetics and Crop Plant Research (IPK, Gatersleben, Germany). The experimental setup involved placing 30 seeds per treatment onto individual germination paper sheets (Keimtestpapier gelb; 120 mm × 300 mm, 160 g/m^2^; Sartorius, Göttingen, Germany). SAP application rates were determined based on manufacturer recommendations. To ensure equivalent water availability per seed across all treatments, polymer quantities were normalized according to their distinct water-absorption capacities (WAC) and the maize thousand-seed weight (TSW = 363 g). Consequently, the calculated application rates were 0.75 g for Merck (470 mL/g WAC), 1.5 g for SWT (250 mL/g WAC), and 173 g for ABG (10 mL/g WAC). Seedlings were germinated for seven days under controlled conditions (25 ± 1 °C, 16/8 h light/dark cycle). Drought stress was induced in the CS and SAP-treated groups by limiting water supply to 5 cc of distilled water per paper sheet for 10 seeds, while CN samples received optimal watering (12 cc of distilled water per sheet). Total RNA was extracted from seedling tissues using the TIANGEN RNAprep Pure Plant Kit, including DNase I digestion, and its integrity was verified with an Agilent 2100 Bioanalyzer. Messenger RNA (mRNA) enrichment was performed using oligo(dT) magnetic beads, followed by fragmentation and conversion into strand-specific cDNA libraries. Libraries underwent standard procedures (end repair, A-tailing, adapter ligation, amplification, and size selection) and were quality-controlled via Qubit fluorometry, real-time PCR, and Bioanalyzer assays by Novogene (Munich, Germany). Sequencing was conducted on an Illumina NovaSeq X Plus platform in paired-end mode. The final dataset comprises 10 samples, split into 13 quality-controlled FASTQ files, with each of the five treatments equally represented, and is publicly available in ENA under accession number ERP180397. For a detailed overview, see Table 1.

**Table 1:** Overview of mRNA-seq FASTQ datasets from maize seedlings subjected to seed coating with SAPs under drought stress

Label	Name of data file/data set	File types (file extension)	Data repository and identifier (DOI or accession number)
Data file 1	MCN102	FASTQ (.fastq.gz)	http://identifiers.org/ena.embl:ERR15607452 [17]
Data file 2	MCN103	FASTQ (.fastq.gz)	http://identifiers.org/ena.embl:ERR15607450 [18]
Data file 3	MCS201	FASTQ (.fastq.gz)	http://identifiers.org/ena.embl:ERR15607456 [19]
Data file 4	MCS202_1^*^	FASTQ (.fastq.gz)	http://identifiers.org/ena.embl:ERR15607453 [20]
Data file 5	MCS202_2^*^	FASTQ (.fastq.gz)	http://identifiers.org/ena.embl:ERR15607454 [21]
Data file 6	MMERCK502	FASTQ (.fastq.gz)	http://identifiers.org/ena.embl:ERR15607505 [22]
Data file 7	MMERCK503	FASTQ (.fastq.gz)	http://identifiers.org/ena.embl:ERR15607488 [23]
Data file 8	MSWT301_1^*^	FASTQ (.fastq.gz)	http://identifiers.org/ena.embl:ERR15607510 [24]
Data file 9	MSWT301_2^*^	FASTQ (.fastq.gz)	http://identifiers.org/ena.embl:ERR15607569 [25]
Data file 10	MSWT302	FASTQ (.fastq.gz)	http://identifiers.org/ena.embl:ERR15607568 [26]
Data file 11	MABG401_1^*^	FASTQ (.fastq.gz)	http://identifiers.org/ena.embl:ERR15607447 [27]
Data file 12	MABG401_2^*^	FASTQ (.fastq.gz)	http://identifiers.org/ena.embl:ERR15607449 [28]
Data file 13	MAB402	FASTQ (.fastq.gz)	http://identifiers.org/ena.embl:ERR15607448 [29]

*Multiple raw data files associated with a single biological replicate represent sequencing lane splits. Reads from these files were merged prior to analysis to represent a single biological sample

## Limitations

Although this study provides important initial insights into maize transcriptomic responses to SAPs under drought conditions, several key areas highlight promising directions for future research. Firstly, this investigation focused on a single maize cultivar (due to foundational restrictions). Given the vast genetic diversity of maize and in line with prior research highlighting variety-specific responses to SAPs [30], incorporating multiple genotypes into subsequent analyses would significantly improve the generalizability of our findings and reveal valuable genotype-specific responses to SAP-mediated drought mitigation. Secondly, this study was limited to the germination seedling development stage (7 days post-germination). Future research should extend to later vegetative and reproductive phases, as SAP effects on plant development and stress resilience may vary significantly across different growth stages. Thirdly, while the current experiment used two biological replicates per treatment group, which were sufficient for detecting major transcriptional shifts, future studies could further increase statistical power and resolution by increasing the number of replicates. This would provide even more robust and reliable insights into subtle regulatory patterns and differential gene expression. Furthermore, in this study, we evaluated three SAP formulations: two fossil-based and one natural-based. While these selections enabled meaningful comparisons across chemical origins, they do not capture the full diversity of available SAP chemistries. Broader screening of additional polymer types will be crucial for elucidating shared and unique molecular effects, ultimately informing the design of crop-specific and environmentally optimized SAP applications.

## Data Availability

The data described in this Data note can be freely and openly accessed in the European Nucleotide Archive (ENA ) under ERP180397 (Table 1).
